# Public perspective toward extended community pharmacy services in sub-national Saudi Arabia: An online cross-sectional study

**DOI:** 10.1371/journal.pone.0280095

**Published:** 2023-10-05

**Authors:** Khalid S. Alghamdi, Max Petzold, Ashraf A. Ewis, Mahdi H. Alsugoor, Khalid Saaban, Laith Hussain-Alkhateeb

**Affiliations:** 1 School of Public Health and Community Medicine, Institute of Medicine, Sahlgrenska Academy, Gothenburg University, Gothenburg, Sweden; 2 Faculty of Clinical Pharmacy, Al Baha University, Al Baha, Saudi Arabia; 3 College of Health Sciences, Umm Al Qura University, Al Qunfudah, Makkah Province, Saudi Arabia; 4 Compliance Department, Al Baha Health Affairs, Al Baha, Saudi Arabia; Lahore Medical and Dental College, PAKISTAN

## Abstract

**Background:**

In many developed countries, the scope of community pharmacy services has extended to include advanced applications. Unlike traditional practices that focus on pharmaceutical sales, extended community pharmacy services (ECPSs) are patient-centred and typically offered by specialised healthcare centres, which improve public health, reduce pressure imposed on healthcare professionals, and rationalise health system expenditures. However, based on the findings of several studies, community pharmacies (CPs) only provide marginalised services. Public reviews are thus crucial to effectively utilise such services. This study explored CPs use among the Saudi public in terms of knowledge, attitudes, and barriers to ECPSs.

**Materials and methods:**

We conducted a cross-sectional web-based survey of a non-probability sample between October and December 2021. Numerical and graphical descriptive statistics were employed with an additional analytical assessment using binary logistic regression to determine the association between participant characteristics and the barriers to ECPSs use.

**Results:**

A total of 563 individuals participated in this study, approximately 33% of which revealed CPs as the first place they visit for medication concerns. Most individuals were not aware of medication therapy management and health screening services (77% and 68%, respectively). Pharmacy clinics offering private counselling and receiving patient electronic medical records were unknown to the participants (78% and 63%, respectively). A substantial proportion of the cohort considered lack of privacy (58%) and inadequate communication with community pharmacists (56%) as key barriers to the use of ECPSs. Logistic regression analysis revealed that the underdeveloped infrastructure of CPs was significantly associated with almost all factors.

**Conclusion:**

Most services and facilities were found to be underutilised. Positive public attitudes were associated with concerns regarding privacy and cost of services. Consistent with Saudi Vision 2030, supporting CPs and increasing the public awareness of ECPSs have significant implications on public health.

## Introduction

Pharmaceutical care can directly and comprehensively address public health needs. The contribution of pharmacists to the care of individuals ensures optimised medicine use and improved health outcomes [1]. Through this approach, community pharmacies (CPs) can assist other healthcare professionals in the dissemination of adequate services, with a focus on patient-centred care [2]. These CP services are categorised into “essential” and “advanced” services by the United Kingdom National Health Services (NHS). Essential services focus on medication dispensing, whereas advanced services include extended community pharmacy services (ECPSs) in addition to routine essential services [3, 4]. ECPSs may be applied via CPs to offer health services usually provided by other healthcare professionals (e.g., general practitioners and nurses) [5]. These services may include vaccinations for seasonal flu, smoking cessation programs, health screening (e.g., blood pressure level, blood glucose level, lipid level, and body weight measurement), and medicine use review (medication therapy management) [4].

The infrastructure of CPs must be well-equipped to ensure efficient service delivery. In this study, a CP facility refers to any equipment that professionally facilitates CP practices. These facilities include automated medication dispensing, electronic prescriptions, medical record systems and health screening devices. In addition, availability of a pharmacy clinic (closed area inside CPs) for private counselling and documented regular follow-ups.

Based on published reports, the mortality and morbidity rates caused by chronic noncommunicable diseases can be reduced by early detection through health screening and regular follow-ups in CPs [5–7]. Therefore, ECPSs serve as a more comprehensive approach that aims to improve public health outcomes, reduce the burden on other primary care settings, and rationalise health system expenditures [8]. Nevertheless, the pharmaceutical care framework assumes a successful patient-pharmacist partnership. This fundamental framework is built on care, trust, communication, and coordination [9]. Furthermore, patients with long-term health conditions, which require regular visits for healthcare management, are more amenable to receiving care through CPs than a physician’s office, primarily due to accessibility and ease of use [10].

Despite the vast array of ECPSs, these services remain underutilised by many patients who rely on essential services. Several studies highlight the need for further assessments of the public’s knowledge and the use of ECPSs, which are minimally available in some developed countries [11]. For example, the pharmaceutical care concept in low- and middle-income countries must be better defined, including the challenges incurred when implementing ECPSs [12]. Serval barriers are associated with these limited services; these may include a smaller number of CPs, the influence of physicians, low public awareness or acceptance, and the absence of an appropriate reimbursement system for ECPSs [13].

In Saudi Arabia (SA), pharmacy is considered a professional career path despite mounting evidence suggesting that CPs are not well equipped to deliver such services [14]. Nevertheless, research on the general public’s view of the role of CPs remains lacking in SA [15], which hinders effective public health policymaking. Therefore, this study aimed to explore the perceptions of the public as users of CPs in SA, with an emphasis on a) identifying patterns of CP utilisation, b) describing knowledge, attitudes, and the degree of importance of selected ECPSs and CP facilities, and c) determining any perceived barriers that could prevent individuals from utilising ECPSs.

## Materials and methods

### Study design and survey instrument

This survey was of a cross-sectional nature and was conducted using an online self-administered questionnaire. A literature search was performed to identify relevant research articles. Moreover, a modified questionnaire was developed using pre-validated survey items to adequately align with the Saudi context [5, 7, 16, 17]. The ECPSs included in this study were identified based on a previously published international systematic review of expanded practice in CPs [18].

Three local staff members from multiple disciplines in public health, pharmacy, and nursing revised the face and content of the survey for validity. The final version was translated to Arabic by an expert translator, using forward-backward methods, until an agreement was reached. The questionnaire was pilot tested using 33 participants who were not part of the main study to ensure that all questions were clear. The feedback from these participants was used to make amendments before the final distribution of the questionnaire. Internal consistency was measured using the Cronbach’s alpha test, which resulted in an acceptable reliability coefficient of 0.78 (non-standardised items).

The electronic survey included a brief introduction to the aims of the study. The survey comprised the following five domains and included 18 closed-ended items: a) characteristics of participants, b) patterns of CP utilisation, c) knowledge of ECPSs and CP facilities, including the degree of importance, d) attitudes toward ECPSs, and e) barriers to ECPSs use (Pages 2–6 in S1 File).

### Sample recruiting and study setting

According to the 2019 Saudi General Authority for Statistics, the total population of Saudi Arabia is approximately 34 million, with residents occupying 13 provinces [19]. Of the population, approximately 10 million live in the targeted regions of Al-Baha, Makkah, and Najran. Therefore, the nonprobability convenience sampling technique was adopted based on the sample size estimate generated by an online calculator (Raosoft Inc.), which employed the following formula:

$$\text{x}=\text{Z}\left(\text{c}/100\right)2\text{r}\left(100-\text{r}\right)\;\text{and}\;\text{n}=\text{N}\;\text{x}/\left(\left(\text{N}-1\right)\text{E}2+\text{x}\right)$$

Where Z (c/100) is the critical value for the confidence level c (5%), r is the fraction of interesting responses (50%), and N is the population size. The margin of error (E) was calculated using the following equation:

$$\text{E}=\text{Sqrt}\left[{}^{\left(N-n\right)x}{/}_{n}{}_{\left(N-1\right)}\right]$$

The recommended sample size was 385 participants [20]. All individuals who spoke Arabic, were older than 18 years, and who used CPs were included in this study.

Owing to the COVID-19 pandemic restrictions imposed in 2021, an online approach using the SurveyMonkey platform was adopted. An electronic link to the survey was distributed via the WhatsApp messenger application, and a snowball recruitment technique was used by asking participants to recruit additional participants over three months, from 1 October 2021 to the end of December 2021. The electronic survey was only limited to one response to prevent duplicate responses.

### Data management and statistical analysis

All collected data were cleaned, coded, and analysed using Stata, version 17. Numerical and graphical descriptive statistics were used. Frequencies and percentages were used to illustrate the characteristics of participants, patterns of CP utilisation, knowledge of ECPSs and CP facilities, and barriers to ECPSs use. In contrast, the degree of importance and attitudes were measured using a five-point Likert scale and collapsed into a three-point Likert scale to more clearly describe the public’s responses. Furthermore, the degree of importance was scored as follows: extremely important/important = 3, neutral = 2, and not important at all/not important = 1. Public attitudes were scored as follows: strongly agree/agree = 3, neutral = 2, and strongly disagree/disagree = 1.

Multiple logistic regression was employed to explore the association between the participants’ characteristics and the key barriers to utilising the services. The barriers were measured as percentages and later transformed into binary outcomes to fit the probability estimates using logistic regression. For instance, respondents who selected the lack of a pharmacy clinic as a barrier were converted to (1) and those who did not select this option were assigned null (0). Crude and adjusted logistic regression modelling and the likelihood ratio test of a forward-wise model-building approach were used, and all tests maintained ≤ 5% as a significant level [21]. All potential confounders from the background and characteristics were assessed using the model.

### Ethics statement

Permission to conduct this study was obtained from the Ethical Research Committee of the Saudi Ministry of Health (MOH), Riyadh (Central IRB log No.21-56E, June 2021). The respondents provided written consent for their responses to be used anonymously and confidentially. The written consent form contained six statements that were read by all participants (Page 1 in S1 File).

## Results

### Characteristics of the participants

A total of 563 participants consented to participate in this study. Among them, 68% were males and 96% were Saudi citizens. Most respondents were married (66%), and most belonged to the 36–45 year old group (32%). More than half of the study cohort had a university degree (55%); 64% were employed and 52% earned ≤ 12000 in Saudi Riyal per month. Regarding health conditions, 2% of the participants had special needs, 26% were current or previous smokers, and 31% had at least one chronic disease. Only 26% of the participants had health insurance. The participant characteristics are presented in Table 1.

**Table 1:** Characteristics of participants in sub-national Saudi Arabia, N = 563.

Variables	Frequency	(%)
**Gender**		
Male	384	(68.21)
Female	179	(31.79)
**Citizenship**		
Saudi	538	(95.56)
Non-Saudi	25	(4.44)
**Marital status**		
Married	374	(66.43)
Single and other *	189	(33.57)
**Age group**		
18–25	124	(22.02)
26–35	133	(23.62)
36–45	178	(31.62)
≥ 46 *	128	(22.74)
**Educational level**		
≤ High school and Diploma *	136	(24.15)
University degree	311	(55.25)
Postgraduate degree (MSc and PhD) *	116	(20.60)
**Job status**		
Employed	361	(64.12)
Unemployed and Retired *	202	(35.88)
**Monthly income in SR**		
≤ 12000 *	291	(51.69)
12001–18000 *	134	(23.80)
> 18001*	82	(14.56)
Undisclosed	56	(9.95)
**Health insurance**		
Yes	145	(25.75)
No	418	(74.25)
**Special need**		
Yes	9	(1.60)
No	554	(98.40)
**Smoking status**		
Previous and current smokers *	149	(26.47)
Non-smoker	414	(73.53)
**Associated chronic diseases**		
Yes *	172	(30.55)
No	391	(69.45)

* Groups were collapsed.

Abbreviations: SR: Saudi Riyal, MSc: Master of Science, PhD: Doctor of Philosophy.

### Public patterns of CP utilisation

All study participants shared the first place they visited to seek health advice when they experienced a minor ailment (e.g., headache, dyspepsia, acute cough, or influenza). A primary health care centre (PHCC) was the first place visited to receive health advice (29%), followed by CPs (27%).

The participants shared the first place they visited for any medication concern (e.g., doubts about dosage, indications, adverse effects, or how a medication should be used). One-third of the participants would first visit CPs (33%). Interestingly, 19% of the participants used online medical websites for information, whereas 16% visited PHCC.

**Fig 1:**
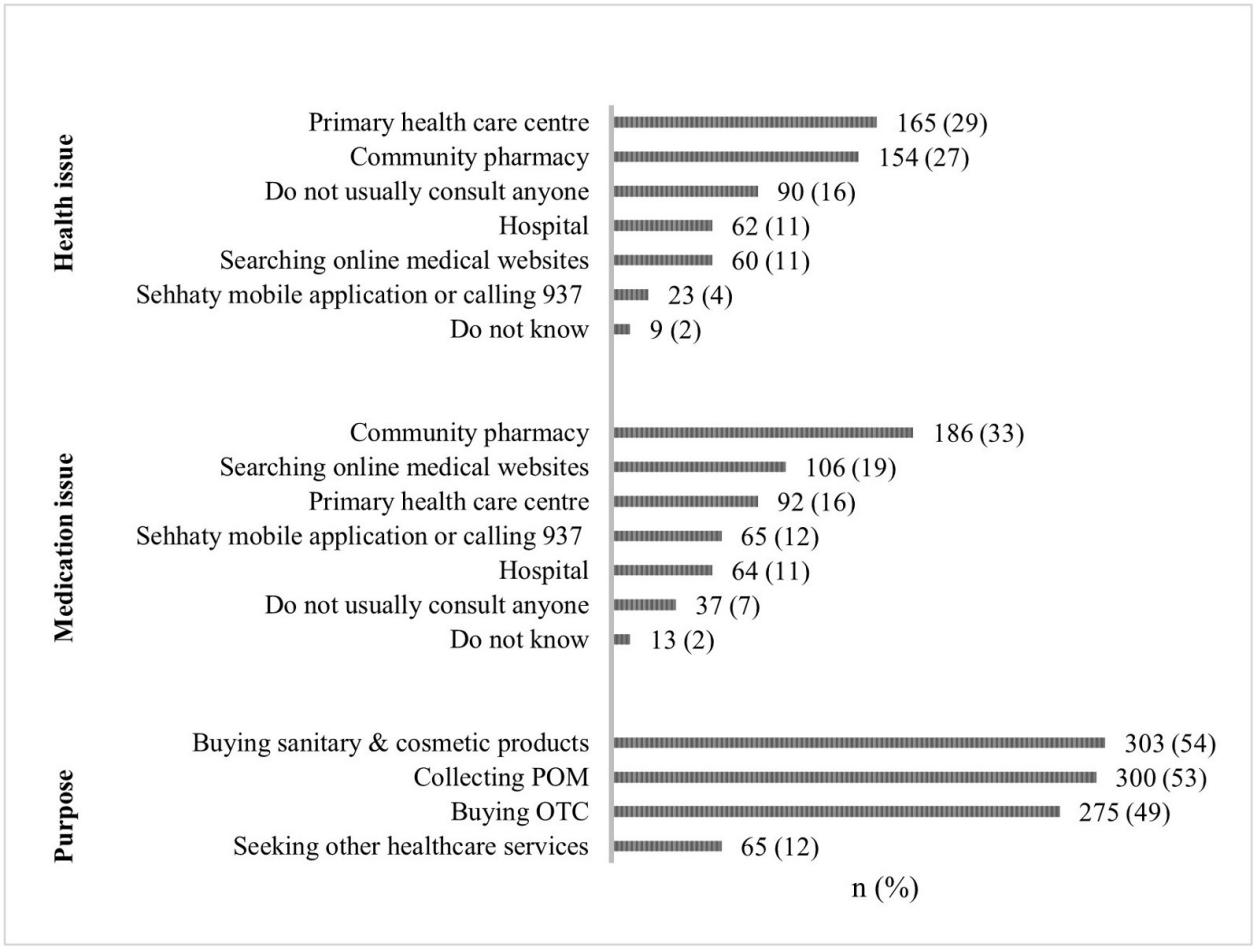
Public pattern of community pharmacy utilisation in sub-national Saudi Arabia, N = 563.

The participants revealed one or more reasons for their visits to CPs in the previous year. Nearly 54% of the participants visited CPs to purchase sanitary and cosmetic products, 53% visited to collect prescription only medicine (POM), 49% visited to purchase over the counter medication (OTC), and only 12% visited to utilise other healthcare services (Fig 1).

### Public knowledge of ECPSs and CP facilities

Of the 563 respondents, 540 (96%) revealed their extent of knowledge regarding ECPSs and CP facilities. Most respondents did not know whether ECPSs existed or utilised the facilities. Among the selected services, travel health programs were known to only 21% of participants, while medication therapy management, health screening, health education and promotion programs, and smoking cessation programs were only known to 23%, 32%, 38%, and 48% of participants. In contrast, most respondents (71%) were aware of the immunisation service.

**Fig 2:**
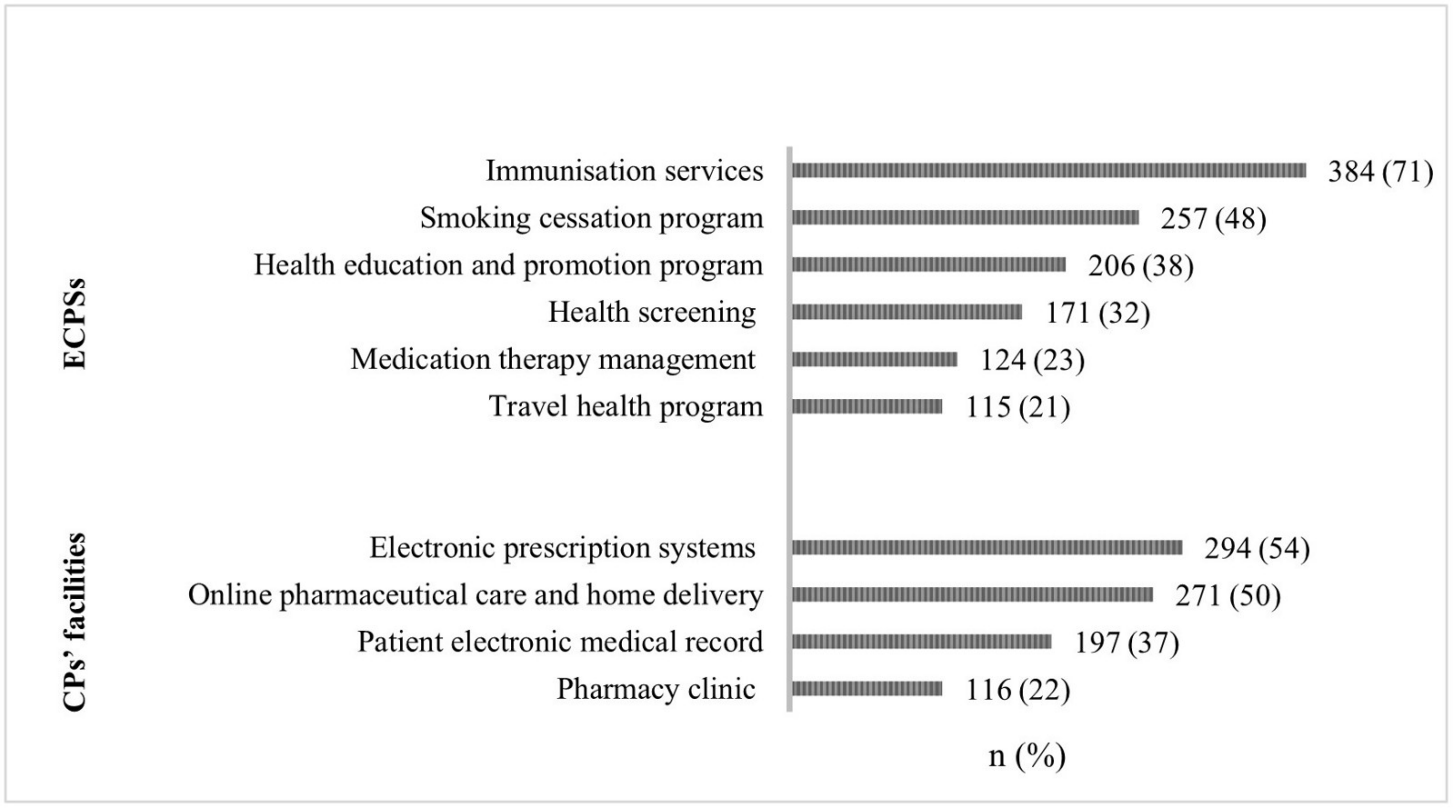
Public knowledge of ECPSs and facilities in sub-national Saudi Arabia, N = 540.

Regarding CP facilities, pharmacy clinics that offer private counselling in a closed area and have access to the electronic medical records of patients were known to only few respondents (22% and 37%, respectively). In contrast, electronic prescription system (e.g., Wasfaty, a national prescription system linked to CPs) and online pharmaceutical care, including prescription delivery, were known by half of the respondents (54% and 50%, respectively; Fig 2).

### Degree of importance of ECPSs and CP facilities

Of the 563 participants, 540 (96%) shared that these services and facilities are critically important. Providing immunisation services as ECPSs and utilising patient electronic medical records at CP facilities were the most important applications according to the public (88% and 86%, respectively). Additional details are provided in Table 2.

**Table 2:** Degree of importance of ECPSs and facilities in sub-national Saudi Arabia, N = 540.

Extended community pharmacy services	Important ^a^ n (%)	Neutral n (%)	Not Important ^b^ n (%)
Immunisation service	473 (87.59)	45 (08.33)	22 (4.07)
Smoking cessation program	471 (87.22)	43 (07.96)	26 (4.81)
Health education and promotion program	465 (86.11)	54 (10.00)	21 (3.89)
Medication therapy management	463 (85.74)	53 (09.81)	24 (4.44)
Health screening service	457 (84.63)	56 (10.37)	27 (5.00)
Travel health program	433 (80.19)	71 (13.15)	36 (6.67)
**Community pharmacy facilities**			
Patient electronic medical record	465 (86.11)	56 (10.37)	19 (3.52)
Online pharmaceutical care and home delivery	465 (86.11)	53 (09.81)	22 (4.07)
Electronic prescription systems (e.g., Wasfaty)	464 (85.93)	52 (09.63)	24 (4.44)
Pharmacy clinic *	443 (82.04)	64 (11.85)	33 (6.11)

The 5-point Likert scales were collapsed into:

^**a**^ (an extremely important/ important), neutral,

^**b**^ (not important at all/ not important).

*A closed area in the community pharmacy for private counselling and regular follow-up.

### Public attitudes toward ECPSs

Of the 563 participants, 535 (95%) shared their attitudes toward ECPSs. Approximately 84% recommended the implementation of selected ECPSs, of which 71% shared that CPs could offer ECPSs that are provided by other healthcare centres, and 66% believe that community pharmacists are adequately qualified to deliver ECPSs. If some of these services require a fee, 31% were willing to pay, whereas 35% were neutral. Table 3 provides more details on the public attitudes toward ECPSs.

**Table 3:** Public attitudes toward ECPSs in sub-national Saudi Arabia, N = 535.

Statements	Agree ^a^ n (%)	Neutral n (%)	Disagree ^b^ n (%)
I recommend ECPSs implementation	447 (83.55)	66 (12.34)	22 (4.11)
CPs could provide ECPSs like other clinics	379 (70.84)	101 (18.88)	55 (10.28)
If any ECPS is implemented, I will register	378 (70.65)	132 (24.67)	25 (4.67)
Pharmacists are qualified to provide ECPSs	351 (65.61)	134 (25.05)	50 (9.35)
If some ECPSs require a fee, I will pay	167 (31.21)	187 (34.95)	181 (33.83)
Current traditional services in CPs are enough	145 (27.10)	147 (27.48)	243 (45.42)

The 5-point Likert scales were collapsed into:

^**a**^ (strongly agree/agree), neutral,

^**b**^ (strongly disagree/disagree).

Abbreviations:

ECPSs: Extended Community Pharmacy Services, CPs: Community Pharmacies.

### Barriers to the use of ECPSs

**Fig 3:**
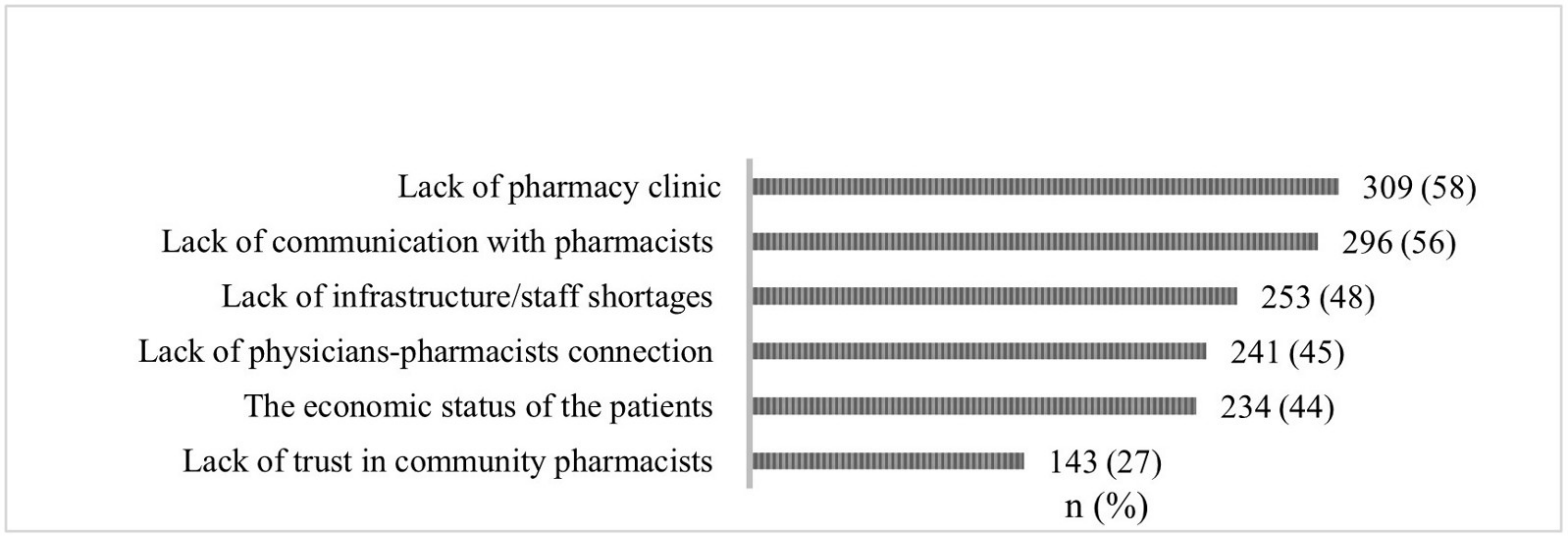
Barriers to the use of extended community pharmacy services in sub-national Saudi Arabia, N = 533.

Of the 563 participants, 533 (95%) selected at least one or more barriers that may hinder their use of ECPSs. According to more than half of the respondents (58%), the lack of a pharmacy clinic inside CPs for private counselling is a key barrier. Further, half of the respondents (56%) shared that they may not use ECPSs as they are not able to communicate adequately with the community pharmacists. Nearly half of CP users (48%) believe that the underdeveloped infrastructure of CPs and staff shortages serve as barriers to these services. Other participants (45%) believe that the lack of an interprofessional connection between physicians and community pharmacists to discuss health conditions is a barrier to the use of ECPSs. The economic status of patients served as another barrier (44%). Only 27% of the respondents considered a lack of trust in the community pharmacists as a barrier. Fig 3 shows the barriers arranged based on the highest to the lowest rating.

According to the adjusted logistic regression analysis, only four barriers were statistically associated with the respondents’ multiple backgrounds and characteristics. No significant association was found between the lack of physician–community pharmacist connections and patient–community pharmacist communications; hence, no such reports were recorded.

The lack of pharmacy clinics was only significantly associated with age group and job status. Moreover, the 26–35 age group was recognised as a barrier, whereas the unemployed or retired groups were not identified as a barrier. Some characteristics of the participants were not significantly associated with this barrier but are noteworthy. For example, females, individuals with higher education, previous or current smokers, and those who do not usually seek health advice for minor ailments are more likely to be concerned about their privacy in the absence of pharmacy clinics.

An underdeveloped CP infrastructure, including staff shortages, was significantly associated with almost all participant characteristics. Females, unmarried, unemployed or retired, and those with no health insurance were less likely to consider this barrier. In contrast, those older than 26 years, highly educated, previous or current smokers, and those who usually do not seek health advice for minor ailments were more likely to consider it as a barrier. Other participant characteristics were not found to be significantly associated with this barrier but are notable. For instance, individuals with higher incomes, special needs, and chronic diseases are more likely to consider this barrier.

Economic status was only significantly associated with marital status, smoking habits, and chronic diseases. Unmarried individuals were less likely to consider their economic status a barrier, whereas previous or current smokers and individuals with chronic diseases were more likely to consider it as a barrier. Despite the lack of a significant association with other characteristics of participants, the following individuals were more likely to consider the cost of ECPSs: the ≥ 26 years age groups, unemployed or retired, and individuals with special needs.

The lack of trust in community pharmacists was significantly associated with gender, monthly income, smoking habits, and the first place to visit for health or medication issues. Moreover, females and individuals who first visit the PHCC to seek health advice were more likely to trust community pharmacists, while individuals with high incomes, previous or current smokers, and those who first visit the hospital for medication concerns were less likely to trust community pharmacists. Other backgrounds and characteristics were not significant but displayed the same trend, including the 26–45 years age group, individuals with ≥ university degree, chronic diseases, and those who do not usually seek health advice for a minor ailment.

Table 4 shows the analytical assessment of barriers to the use of ECPSs based on different backgrounds and characteristic factors.

**Table 4:** Crude and adjusted logistic regression of the association between barriers to ECPSs use and characteristics of participants in sub-national Saudi Arabia, N = 533.

Variables *(Reference)*	Lack of pharmacy clinic		Infrastructure/ staff shortage		Patient’s economic status		Lack of trust in pharmacists	
	COR (95% CI)	COR (95% CI)	COR (95% CI)	AOR (95% CI)	COR (95% CI)	AOR (95% CI)	COR (95% CI)	AOR (95% CI)
**Gender** *(Males)*								
Females	1.24 (0.86–1.79)	1.24 (0.85–1.78) ^a^	**0.61 (0.42–0.90)**	**0.61 (0.42–0.90)** ^a^	0.80 (0.52–1.10)	0.75 (0.52–1.09) ^a^	**0.63 (0.41–0.98)**	**0.63 (0.41–0.97)** ^a^
**Marital status** *(Married)*								
Single and other *	1.17 (0.81–1.68)	0.94 (0.63–1.40) ^b^	**0.60 (0.42–0.86)**	**0.58 (0.40–0.84)** ^**g**^	**0.70 (0.46–0.96)**	**0.67 (0.46–0.96)** ^d^	0.87 (0.60–1.30)	0.87 (0.57–1.32) ^d^
**Age group** *(18–25)*								
26–35	**1.81 (1.08–3.01)**	**1.81 (1.08–3.02)** ^a^	**1.68 (1.00–2.80)**	**1.70 (1.01–2.84)** ^a^	1.18 (0.72–1.96)	1.18 (0.71–1.96) ^a^	1.20 (0.70–2.00)	1.16 (0.66–2.03) ^a^
36–45	1.20 (0.75–1.92)	1.23 (0.77–1.97)	**2.30 (1.42–3.73)**	**2.47 (1.51–4.04)**	1.21 (0.80–2.00)	1.25 (0.77–2.02)	1.30 (0.80–2.15)	1.35 (0.79–2.29)
≥ 46 *	1.31 (0.77–2.18)	1.29 (0.77–2.16)	**2.10 (1.24–3.60)**	**2.03 (1.20–3.46)**	1.10 (0.70–1.87)	1.09 (0.64–1.83)	0.71 (0.40–1.32)	0.69 (0.37–1.27)
**Educational level** *(****≤*** *Diploma)* *								
University degree	0.98 (0.64–1.49)	0.92 (0.61–1.42) ^b^	0.83 (0.54–1.26)	0.86 (0.56–1.32) ^a^	0.90 (0.60–1.38)	0.90 (0.59–1.38) ^d^	1.23 (0.80–2.00)	1.24 (0.76–2.04) ^d^
Postgraduate degree (MSc, PhD) *	1.57 (0.89–1.81)	1.32 (0.76–2.28)	**1.72 (1.03–2.87)**	**1.70 (1.02–2.86)**	0.93 (0.60–1.60)	0.90 (0.53–1.50)	1.40 (0.80–2.50)	1.33 (0.74–2.40)
**Job status** *(Employed)*								
Unemployed and retired *	**0.60 (0.42–0.86)**	**0.61 (0.43–0.87)** ^a^	**0.58 (0.41–0.84)**	**0.60 (0.41–0.86)** ^a^	1.00 (0.71–1.50)	1.24 (0.83–1.84) ^e^	0.80 (0.51–1.16)	0.83 (0.55–1.26) ^d^
**Monthly income in SR** *(12001–18000)* *								
≤ 12000 *	0.69 (0.45–1.06)	0.83 (0.52–1.30) ^b^	**0.60 (0.39–0.92)**	0.64 (0.41–1.00) ^d^	0.76 (0.50–1.20)	0.80 (0.52–1.24) ^d^	0.90 (0.54–1.42)	1.0 (0.60–1.64) ^d^
> 18001 *	1.04 (0.58–1.87)	1.07 (0.60–1.92)	1.24 (0.70–2.19)	1.30 (0.73–2.31)	0.73 (0.42–1.30)	0.75 (0.43–1.33)	**1.91 (1.10–3.50)**	**2.11 (1.14–3.88)**
**Health insurance** *(insured)*								
Non insured	0.93 (0.63–1.38)	0.96 (0.64–1.42) ^b^	0.66 (0.45–1.00)	**0.63 (0.41–0.96)** ^c^	1.10 (0.72–1.56)	1.09 (0.74–1.62) ^d^	0.70 (0.46–1.07)	0.73 (0.48–1.13) ^d^
**Special need** *(Non)*								
Yes	0.97 (0.21–4.36)	1.03 (0.23–4.67) ^c^	1.48 (0.33–6.70)	1.59 (0.34–7.24) ^c^	1.71 (0.38–7.74)	1.78 (0.39–8.12) ^e^	0.50 (0.10–3.80)	0.37 (0.04–3.25) ^d^
**Smoking status** *(Non)*								
Previous and current smokers *	1.24 (0.84–1.85)	1.18 (0.79–1.76) ^b^	**1.70 (1.13–2.50)**	**1.67 (1.10–2.54)** ^c^	**1.60 (1.10–2.36)**	**1.60 (1.07–2.35)** ^e^	**2.30 (1.5–3.44)**	**2.14 (1.38–3.31)** ^c^
**Chronic disease** *(Non)*								
Yes *	0.78 (0.54–1.14)	0.82 (0.55–1.22) ^c^	1.20 (0.83–1.74)	1.21 (0.83–1.77) ^b^	1.10 (0.76–1.60)	**1.11 (1.08–2.36)** ^d^	1.23 (0.81–1.85)	1.26 (0.82–1.91) ^d^
**The first place to visit seeking health advice** *(Other options)* *								
PHCC	0.93 (0.63–1.36)	1.00 (0.66–1.43) ^b^	0.78 (0.53–1.14)	0.72 (0.60–1.31) ^h^	0.92 (0.63–1.35)	0.89 (0.61–1.31) ^a^	**0.60 (0.37–0.93)**	**0.55 (0.35–0.88)** ^a^
Don’t usually consult anyone	1.25 (0.77–2.04)	1.25 (0.77–2.04)	**1.84 (1.14–2.97)**	**1.95 (1.20–3.15)** ^f^	1.15 (0.72–1.85)	1.17 (0.73–1.88) ^e^	1.38 (0.83–2.30)	1.40 (0.83–2.31) ^a^
**The first place to visit asking about medication** *(Other options)* *								
Hospital	0.84 (0.48–1.47)	0.84 (0.48–1.47) ^b^	0.91 (0.53–1.59)	0.88 (0.50–1.54) ^e^	1.00 (0.57–1.73)	0.96 (0.55–1.69) ^e^	**2.00 (1.13–3.54)**	**2.12 (1.19–3.78)** ^d^

Barriers to the lack of physicians-community pharmacists connection and lack of patients-pharmacists communication are not shown in this table due to the lack of a significant association.

* Groups were collapsed.

**Bold** indicates significance at P value ≤ 0.05

Abbreviations: COR: Crude Odd Ratio, AOR: Adjusted Odd Ratio, CI: Confidence Interval, PHCC: Primary Health Care Centre.

^a^, adjusted for standard characteristics (sex/ age).

^b^, adjusted for job status.

^c^, adjusted for income.

^d^, adjusted for smoking status.

^e^, adjusted for marital status.

^f^, adjusted for health insurance.

^h^, adjusted for the first place to visit for health advice: the hospital.

## Discussion

The concept of pharmaceutical care has undergone a significant transition from its traditional model, which is merely concerned with dispensing practices, to a more interprofessional model with broader but crucial public health objectives. By 2030, SA will welcome two major events with national and global agendas. The first event is the assessment of the Sustainable Development Goals (SDGs), where target 3.4, “to reduce by one-third premature mortality from noncommunicable diseases through prevention and treatment” [22], will be assessed. The second major event is the completion and evaluation of the Saudi 2030 National Vision projects [23]. Both events have played a significant role in driving the current health system reengineering process in SA. Hence, the changes anticipated for CP practices will essentially act as substantial factors from the perspective of the public, who are the receivers of ECPSs, and can be translated into a practical pharmaceutical care approach.

In this study, all respondents were CP customers, including patients. Almost one-third of the cohort shared that CP was the first place visited for health advice regarding a minor ailment. Published reports from other sources revealed a broad range of this pattern, such as in Ethiopia (30%) [8], Portugal (36%) [19], Malaysia (46%) [24], Kuwait (61%) [25], and Qatar (91%) [9], which may reflect different social, health systems, and economic aspects. Despite the high access rate to PHCC in SA owing to the free-of-charge health system model, the role of CPs in providing timely, easy, and relatively cheap medical advice can support the country’s medical services in their response to more severe health conditions.

The public also visited CPs to collect POM and OTC; almost half of the participants practised self-medication or collected medications prescribed by physicians from CPs. This general tendency was consistent with the findings of both national and international studies [26–30]. Notably, 12% of the study respondents visited CPs for other healthcare services, such as ECPSs. Only minimal services could explain this small proportion of interest eventually reaching the public, which is supported by the low knowledge of respondents regarding ECPSs, as revealed in this study.

Most respondents needed to know that CPs could provide medication therapy management, health screening, body weight management, smoking cessation services, and other services. The lack of ECPSs in local CPs might be due to three key factors: a) high standards and requirements set by the Saudi MOH against ECPSs implementation (e.g., medication therapy management and health screening) [31], b) low functionality or professionality of CPs, and c) low public awareness due to low priority for integrating ECPSs in wider public communication platforms. The latter factor is similar to other settings reported elsewhere. Although ECPSs have been available in the United Kingdom since 2005, the public still needs to be aware of CPs’ role [32].

During the COVID-19 pandemic, the Saudi MOH signed a memorandum with one of the largest chain CPs to deliver COVID-19 vaccines [33]; however, the distribution was limited to large provinces in SA. The capacity and readiness of CPs in these provinces for the assigned services may be higher than those in small provinces. Raising public health awareness among people is a crucial government initiative, and may explain the high proportion of public knowledge regarding the availability of immunisation services among Saudi CPs.

The Saudi MOH has launched contact channels for the public. Moreover, a free 24-hour call centre was established in 2016 to obtain public concerns regarding any health issue, followed by the e-health application, “Sehhaty”, to facilitate visual medical consultations on smartphones [34, 35]. However, the results reveal a deficient proportion of these applications. The participants tended to search for websites instead of utilising the MOH channels. Long queues for these channels or low public awareness may explain this low proportion.

In 2018, the Saudi MOH released the Wasfaty system in multiple stages using a national electronic prescription platform through contracted CPs [36]. Notably, nearly half the participants were aware of this platform. Additionally, electronic medical records are inaccessible to local CPs. However, two-thirds of the public have yet to experience this facility. In 2010, Al Hassan et al. called for the development of guidelines or regulations for professional practice related to patients’ medical records [37]. However, this facility is only available in a few other countries [38]. Linking patient medical history to CPs through a regulated platform would efficiently facilitate the modern digitalised form of enhanced physician-pharmacist-patient communication, which can have significant implications for public health and healthcare delivery.

Based on the study results, the public strongly supports the implementation of ECPSs in CPs with a high degree of importance, a positive attitude, and a desire to support the future scaling-up of such services. This attitude is consistent with the results of several global studies [5, 11, 12, 32, 39]. However, there are several barriers and public concerns regarding these services. Against the direct assessment of the SDGs target 3.8, “achieving universal healthcare access” [40], barriers to the use of ECPSs were processed through in-depth logistic regression analysis. In public opinion, the infrastructure of CPs, including the healthcare workforce allocated to these services, is important. Almost all respondents’ backgrounds and characteristic factors (socio-demography, economy, health status, and behaviours) were associated with the underdeveloped infrastructure of CPs and staff shortages, which served as key barriers.

The lack of pharmacy clinics to deliver private counselling, immunisation service, and regular follow-up was a considerable barrier, as most respondents did not receive confidential ECPSs inside CPs. This finding is consistent with previous national studies that highlighted public privacy and confidentiality concerns [41–45]. A systematic international review of feedback from CPs users by Anderson et al. considered privacy and confidentiality during advising sessions as critical factors in increasing the acceptability of the roles of CPs [46]. Furthermore, male community pharmacists dominated the private sector in SA (89%) [47]. This predominancy corresponds to the higher probability of females pleading a lack of privacy due to the unavailability of pharmacy clinics. This issue has been addressed elsewhere, where 58% of Saudi females favour private counselling by female pharmacists [48]. Hence, female pharmacists need encouragement and support to work in CPs. Additionally, other participants from different backgrounds expressed concerns regarding their privacy. Therefore, Saudi CPs must be better designed and equipped with good facilities for a safe and pleasant environment where pharmacist-patient can exchange sensitive information.

The economic status of the respondents was an additional barrier associated with several variables in this study. In fact, two-thirds of the study respondents disagreed or were neutral for paid ECPSs. Notably, only 25% of the public had health insurance. Securing health insurance encourages the population to undergo medical check-ups in the Saudi private health sector [49]. In SA, all private sector employees and their family members are health insured by law. During the performance of this study, a new Saudi law was introduced. This law grants full health insurance to government employees and their families, covering private sector healthcare services. However, not all government agencies provide health insurance to employees. Therefore, health insurance coverage remains limited to agencies permitted by labour regulations [50]. This economic barrier should be considered essential when universal health insurance coverage or remuneration for the ECPSs are lacking.

Although 73% of the study participants expressed their trust in community pharmacists, a lack of patient-community pharmacist communication was found. More than half of the respondents considered this lack of communication as a potential barrier. Workload and staff shortages in the absence of scheduled appointments in CPs can lead to poor communication with community pharmacists. Notably, the lack of interprofessional connections between physicians and community pharmacists to enable a discussion of patients’ health conditions was considered by the participants. Evidence of a physician-pharmacist collaboration in clinical settings has been demonstrated to improve public health outcomes [39, 51]. However, there is a lack of social and professional acceptance of community pharmacists among physicians in SA [52]. These factors affect the overall perspective of pharmaceutical care.

### Limitations

The sampling and design of this study were affected by strict COVID-19 distancing regulations. Relying on a self-administered web-based questionnaire as a source of information in a cross-sectional and using non-probability sampling technique due to COVID-19 pandemic restrictions study can raise concerns regarding the interpretation and generalisation of the findings, which were strongly considered prior to drawing any conclusions. Moreover, this approach unintentionally excluded CP users who do not use web-based platforms. Additionally, this data collection technique neglects the physical observation of the actual situation of services delivery to the public. Most respondents had higher degrees, which may have impacted their attitudes toward ECPSs evaluation. Further, elderly participants, females, people with associated chronic diseases, people with special needs, and those with health insurance were under-represented in the cohort. Some responses were found to be skewed, as noted for a few outcome categories. Nevertheless, despite its non-probabilistic nature, the integration of the snowball sampling approach enhanced the response rate and added value to the questionnaire distribution via web applications. The descriptive nature of the study design, coupled with a careful and thorough multiple regression process, adjusts for the associated issues attributed to the imbalanced distribution of some groups.

## Conclusion

This study is timely due to upcoming national and global agendas and will likely provide valuable findings to guide future policies. Based on the findings, ECPSs and CP facilities need to be adequately utilised by the public, despite the public’s appreciation of these service implementation. The fundamental aspects perceived by the public relate to their privacy, cost of services, and lack of professional communication where such ECPSs are provided. However, some groups are at a higher risk of being exposed to these barriers, particularly females, individuals with low income, those with special needs and non-health insured. CPs must be better designed and equipped with facilities for effective pharmacy practices.

## Supporting information

S1 File Public questionnaire (English and Arabic versions). (PDF)

S2 File Data. (CSV)
