# Supplementary material for: Public perspective toward extended community pharmacy services in sub-national Saudi Arabia: An online cross-sectional study
Source: PLoS One. 2023 Oct 5;18(10):e0280095. doi: 10.1371/journal.pone.0280095 (PMC10553341; doi:10.1371/journal.pone.0280095)
Supplement: S1 File — (PDF) [file pone.0280095.s001.pdf]

English Version

Survey

Public perspective toward extended community pharmacy services in sub-national Saudi Arabia: An online cross-sectional study

Introduction of study and participant consent:

Hello,

We are interested in your thoughts about community pharmacy services. We will ask you about your patterns and use of the community pharmacy. Your knowledge and the extended services provided by community pharmacies that you might be aware of and benefiting from so far. Also, your attitude about implementing these extended services to improve public health. Finally, the barriers you might face using these services from your point of view.

Your participation in this questionnaire is invaluable and will help us obtain accurate data on community pharmacy services. However, it is entirely voluntary, and not participating will not affect the service you receive from those pharmacies or any other healthcare institutions. If you agree to participate in the study, please answer each question as honestly as possible. You may skip any question that you do not wish to answer it.

This survey has been revised and approved by the central IRB, Ministry of Health, Riyadh, Saudi Arabia. The survey will take about 5-10 minutes, and your time is highly appreciated. In case of inquiry, you can email us via this email: khalid.alghamdi@gu.se.

Please read the following statements to affirm your agreement:

- I understand that participation in this study is entirely voluntary, and this survey is anonymous.
- I am a citizen or resident of Saudi Arabia.
- I am over 18 years old.
- I have visited the community pharmacy in the past year.
- I give my full consent to participate in this study.

I agree and would like to participate (Please press next to start the survey)

Next

A. Characteristics of participants:

First, we would like to get some general demographics from you. People may have different needs and concerns based on their life experiences and health condition.

1. Participant Gender:

☐ Male ☐ Female

2. Are you a Saudi citizen or a resident?

☐ Saudi ☐ Non-Saudi

3. Marital status:

☐ Married ☐ Single ☐ Other

4. You belong to which age group?

☐ 18─25 ☐ 26─35 ☐ 36─45 ☐ 46─55 ☐ 56─65 ☐ > 65

5. Educational level:

☐ High School or less ☐ Diploma ☐ University degree (BSc /BA)

☐ Master’s degree (MSc/ MA) or equivalent degree ☐ Doctor of Philosophy (PhD) or equivalent degree

6. Job Status:

☐ Employed ☐ Unemployed ☐ Retired

7. Average monthly income in Saudi Riyal:

☐ Less than 3,000 ☐ 3,000 ─ 7,000 ☐ 7,001 ─ 12,000 ☐ 12,001 ─ 18,000 ☐ 18,001 ─ 25,000 ☐ More than 25,000

☐ Undisclosed

8. Do you have health insurance?

☐ Yes ☐ No

9. Do you have a special need?

☐ Yes ☐ No

10. Do you smoke?

☐ No ☐ Current smoker ☐ Previous smoker

11. Do you have any chronic disease?

☐ No, I don’t have any disease

☐ Obesity

☐ Diabetes Mellitus

☐ Blood pressure

☐ Blood lipids (e.g., Cholesterol)

☐ Asthma/allergic asthma

☐ Osteoporosis

☐ Other

B. Patterns of community pharmacies utilisation:

12. What is the first place you seek advice when a minor ailment arises?

(e.g., headaches, dyspepsia, acute cough, influenza)

☐ Primary Health Care Centre

☐ Community Pharmacy

☐ Hospital

☐ Sehhaty Application on your Mobile or Calling 937

☐ I do not usually consult anyone

☐ I do not know

13. What is the first place you go for any medication questions?

(e.g., doubts about dosage, indication, how it is used, adverse effects)

☐ Primary Health Care Centre

☐ Community Pharmacy

☐ Hospital

☐ Sehhaty Application on your Mobile or Calling 937

☐ I do not usually consult anyone

☐ I do not know

14. In the last 12 months, for what purpose did you visit the community pharmacy?

(Multiple responses possible)

☐ To collect prescription-only medicine (POM)

☐ To collect over the counter (OTC) medicine

☐ To purchase sanitary/cosmetic products

☐ To get other healthcare services

C. Knowledge of extended community pharmacy services, facilities and the degree of importance:

Now, we will show you a list of some extended community pharmacy services and some facilities that are probably available at the community pharmacies OR could be available shortly. Regarding each one, can you please tell us if:

15. Do You know or have you heard about it?

**SERVICES AND FACILITIES:**<br>*Description*<br>**Extended community pharmacy services**	**I know/**<br>**I have heard**	**I do not know/**<br>**I have not heard**
**Health screening:**<br>*E.g., Bodyweight management, blood glucose meters, blood pressure meters, cholesterol level, peak-flow meters (for asthma), & osteoporosis screening.*	◯	◯
**Smoking cessation program:**<br>*Counselling, aid, & treatments with regular follow-up.*	◯	◯
**Immunisation:**<br>*E.g., Seasonal flu vaccination service & COVID-19 testing.*	◯	◯
**Travel health program:**<br>*Necessary preventive & therapeutic care before travelling.*	◯	◯
**Medication therapy management:**<br>*E.g., Assessing a patient's health status & formulating a medication treatment plan, monitoring compliance with medicines, reporting medication errors, reporting adverse drug reactions, preventing medication-related problems, & offering feedback to the patient's physician about health condition progress with a treatment plan.*	◯	◯
**Health education and promotion program:**<br>*Improving health literacy, knowledge, & developing life skills in the community.*	◯	◯
**Community pharmacy facilities:**	**I know/**<br>**I have heard**	**I do not know/**<br>**I have not heard**
**Electronic prescription (e.g., WASFATY):**<br>*Linking health care centres & hospitals with community pharmacies to collect your medicines from community pharmacies.*	◯	◯
**Patients electronic medical record:**<br>*Available medical history to follow up on your health condition.*	◯	◯
**Providing online pharmaceutical care and home delivery:**<br>*Providing pharmaceutical care via the website or by phone & deliver your medicines to you.*	◯	◯
**Pharmacy clinic:**<br>*Providing private counselling & regular follow-up in a designated closed area inside the community pharmacy.*	◯	◯

16. How important do you rate each service and facility?

**SERVICES AND FACILITIES:** **Extended community pharmacy services:** *Description*	**Extremely important**	**important**	**Neutral**	**Not important**	**Not important at all**
**Health screening:** *E.g., Bodyweight management, blood glucose meters, blood pressure meters, cholesterol level, peak-flow meters (for asthma), & osteoporosis screening.*	◯	◯	◯	◯	◯
**Smoking cessation program:** *Counselling, aid, & treatments with regular follow-up.*	◯	◯	◯	◯	◯
**Immunisation:** *E.g., Seasonal flu vaccination service & COVID-19 testing.*	◯	◯	◯	◯	◯
**Travel health program:** *Necessary preventive and therapeutic care before travelling.*	◯	◯	◯	◯	◯
**Medication therapy management:** *E.g., Assessing a patient's health status & formulating a medication treatment plan, monitoring compliance with medicines, reporting medication errors, reporting adverse drug reactions, preventing medication-related problems, & offering feedback to the patient's physician about health condition progress with a treatment plan.*	◯	◯	◯	◯	◯
**Health education and promotion program:** *Improving health literacy, knowledge, & developing life skills in the community.*	◯	◯	◯	◯	◯
**Community pharmacy facilities:** *Description*	**Extremely important**	**important**	**Neutral**	**Not important**	**Not important at all**
**Electronic prescription (e.g., WASFATY):** *Linking health care centres & hospitals with community pharmacies to collect your medicines from community pharmacies.*	◯	◯	◯	◯	◯
**Patients electronic medical record:** *Available medical history to follow up on your health condition.*	◯	◯	◯	◯	◯
**Providing online pharmaceutical care & home delivery:** *Providing pharmaceutical care via the website or by phone & deliver your medicines to you.*	◯	◯	◯	◯	◯
**Pharmacy clinic:** *Providing private counselling & regular follow-up in a designated closed area inside the community pharmacy.*	◯	◯	◯	◯	◯

D. Attitudes towards extended community pharmacy services:

Now, your attitude towards these extended services and community pharmacists is critical.

17. Please indicate your level of agreement with each of the following statements:

Statements	Strongly agree	Agree	Neutral	Disagree	Strongly disagree
Community Pharmacies could provide these extended services currently offered by other healthcare units.	○	○	○	○	○
Community pharmacists are qualified to provide professional pharmaceutical care to improve public health outcomes.	○	○	○	○	○
Due to their importance, I recommend extended pharmacy services implementation in community pharmacy settings.	○	○	○	○	○
If some extended pharmacy services program is implemented in community pharmacies, I will register for this program.	○	○	○	○	○
I would pay for these services if some extended pharmacy services require a fee.	○	○	○	○	○
The current services (traditional) provided by community pharmacies are enough to cover the population's needs.	○	○	○	○	○

E. Barriers to using extended community pharmacy services:

Finally, you have an idea and attitude about extended community pharmacy services.

18. From your point of view, what are the most barriers preventing you from using these extended services?

You may choose more than one option.

☐ Inability to communicate adequately with the community pharmacists. (e.g., overcrowding inside the pharmacy or no previously scheduled appointment service).

☐ Lack of pharmacy clinic (a designated private counselling area inside the pharmacy to discuss any health condition).

☐ Lack of trust in community pharmacists to deliver extended community pharmacy services.

☐ Lack of connection between physicians and community pharmacists regarding a patient's health condition.

☐ Economic status of patients/customers to pay for a specific extended service.

☐ Infrastructure of community pharmacies and qualified staff are insufficient to provide these services.

End of survey

Thank you for participating in our survey. Kindly post this survey link to others: https://www.surveymonkey.com/r/3NFCJF8

(Arabic Version)

(النسخة العربية)

استبيان

وجهات نظر المجتمع تجاه الخدمات الصيدلانية الموسَّعة للصيدليات المجتمعية (الأهلية) في بعض مناطق المملكة العربية السعودية: دراسة مقطعيّة عبر الانترنت

مقدمة موجزة عن الدراسة وإقرار الموافقة على المشاركة:

مرحبا،

نحن مهتمون بآرائك فيما يتعلق بخدمات الصيدليات المجتمعية **(ونقصد بها الصيدليات الأهلية في القطاع الخاص)** ونود أن نطرح عليك بعض الأسئلة حول نمط ترددك على هذه الصيدليات ومدى معرفتك بالخدمات الصيدلانية الموسّعة وبعض المرافق التي تقدمها أو قد تقدمها مستقبلا ومدى أهميتها من وجهة نظرك وموقفك كمستفيد بخصوص توفير هذه الخدمات لتحسين الصحة العامة، بالاضافة الى العوائق التي قد تواجهها في الاستفادة من هذه الخدمات.

تعد مشاركتك في هذا الاستبيان فائقة الأهمية وستساعدنا في جمع بيانات دقيقة حول خدمات الصيدليات المجتمعية الموسَّعة، كما نود أن ننوه بأن المشاركة في هذا الاستبيان تعد تطوعيّة، وعدم مشاركتك، لن يؤثر سلبا على الخدمات التي تتلقاها من هذه الصيدليات أو أيّ من المراكز الصحية الأخرى. وفي حال موافقتك على تعبئة هذا الاستبيان فنرجو منك تحري الدقة في جميع اجاباتك، كما يمكنك تخطي الأسئلة التي لا ترغب في الإجابة عنها.

لقد تمت مراجعة محتوى هذا الاستبيان والموافقة على توزيعه من اللجنة المركزية لأخلاقيات البحوث في وزارة الصحة السعودية والمدة التقريبية لإكمال هذا الاستبيان هي من 5-10 دقائق ونثمن لك وقتك. وفي حالة الاستفسار، يمكنك مراسلتنا من خلال هذا البريد الالكتروني: khalid.alghamdi@gu.se

فضلا، اضغط على ايقونة المشاركة ادناه لتأكيد موافقتك بعد قراءتك لما يلي:

- أعلم أن تعبئة هذا الاستبيان تطوعيّة ولا يكشف عن الهوية.
- زُرت صيدلية مجتمعية خلال السنة الأخيرة.
- أنا مواطن أو مقيم في المملكة العربية السعودية.
- عمري يزيد عن (18) عاما.
- أوافق تماما على المشاركة في هذه الدراسة.

الرجاء الضغط على زر التالي لإكمال الاستبيان

التالي

أ. الخصائص الاجتماعية للمشارك:

بداية، نود معرفة بعض المعلومات التي تتعلق بالتركيبة السكانية المحلية، وذلك لاختلاف حاجة كل شخص وتنوع اهتمامه وطبيعة تجربته وحالته الصحية.

1. الجنس:

☐ ذكر

☐ انثى

2. مواطن أم مقيم؟

☐ سعودي

☐ غير سعودي

3. الحالة الاجتماعية:

☐ متزوج

☐ أعزب

☐ أخرى

4. الفئة العمرية:

☐18-25 ☐ 26-35 ☐ 36-45 ☐ 46-55 ☐ 56-65 ☐ أكبر من 65

5. المستوى التعليمي:

☐ الثانوية العامة أو ما دونها

☐ دبلوم

☐ بكالوريوس

☐ درجة الماجستير أو ما يعادلها

☐ درجة الدكتوراه أو ما يعادلها

6. الحالة الوظيفية:

☐ موظف

☐ غير موظف

☐ متقاعد.

7. متوسط الدخل الشهري:

أقل من 3000 ☐، 3000-7000 ☐، 7001-12000 ☐، 12001-18000 ☐، 18001-25000 ☐، أعلى من 25000 ☐

أفضل عدم الإجابة ☐

8. لديك تأمين طبي؟

☐ نعم ☐ لا

9. من ذوي الاحتياجات الخاصة ؟

☐ نعم ☐ لا

10. هل أنت مدخن ؟

☐ لا ☐ مدخن حالي ☐ مدخن سابق

11. هل تعاني من أمراض مزمنة؟

☐ لا أعاني من أي مرض مزمن

☐ سمنة

☐ سكري

☐ ضغط دم

☐ دهون (كليسترول)

☐ ربو أو حساسية صدر

☐ هشاشة عظام

☐ أخرى

ج. نمط تردد المستفيد على الصيدليات المجتمعية:

12. اين تتجه أولا للحصول على استشارة طبية عند ظهور عرض بسيط (مثل الصداع أو عسر الهضم أو)؟

☐ مركز رعاية صحية اولية أو عيادة طبية

☐ صيدلية مجتمعية

☐ مستشفى

☐ تطبيق صحتي على هاتفك المحمول أو الاتصال بـ 937

☐ لا أستشير أحدا في الغالب

☐ لا أعلم

13. اين تتجه أولا للسؤال عن الأدوية (مثلا: الشك في مقدار الجرعة، أو اعراض دوائية أو ، أو كيفية الاستعمال)؟

☐ مركز رعاية صحية اولية أو عيادة طبية

☐ صيدلية مجتمعية

☐ مستشفى

☐ تطبيق صحتي على هاتفك المحمول أو الاتصال بـ 937

☐ لا أستشير أحدا في الغالب

☐ لا أعلم

14. لأيّ من الأسباب التالية زرت الصيدلية المجتمعية خلال الاثني عشر شهرا الماضية؟ (يمكن اختيار عدة إجابات):

☐ لصرف وصفة طبية

☐ لأخذ دواء لا يستلزم وصفة طبية

☐ لشراء منتجات صحية أو تجميلية

☐ للحصول على خدمات صحية أخرى

د. معرفة المستفيد بالخدمات الصيدلانية الموسَّعة والمرافق وتقييمه لمدى أهميتها:

سنعرض عليك قائمة ببعض الخدمات والمرافق والتي قد تكون متاحة في الصيدليات المجتمعية، أو قد تتاح في المستقبل. ونودّ منك أن تخبرنا بالآتي:

15. هل تعلم بوجود هذه الخدمة أو سمعت عنها سابقا؟

الخدمات الصيدلانية والمرافق ووصف كل خدمة<br><br>الخدمات الصيدلانية الموسّعة:	أعلم عنها<br>أو سمعت عنها	لا أعلم عنها<br>أو لم اسمع عنها
**الفحص الصحي:**<br>*وصف الخدمة: برنامج إدارة ومتابعة الوزن ونمط التغذية الصحي. قياس مستوى السكر، أو قياس مستوى ضغط الدم، أو قياس مستوى الكوليسترول، أو قياس نسبة الاكسجين (لحالات الربو). فحص هشاشة العظام وغيرها.*	○	○
**برنامج الإقلاع عن التدخين:**<br>*وصف الخدمة: برنامج متكامل للمدخنين ويشمل ذلك أدوات المساعدة والعلاجات والاستشارات والمتابعة المستمرة*	○	○
**خدمات التحصين:**<br>*وصف الخدمة: توفير واعطاء اللقاحات، أو لقاحات الانفلونزا الموسمية، أو فحص كوفيد-19.*	○	○
**برنامج صحة المسافر:**<br>*وصف الخدمة: رعاية وقائية، أوعلاجية قبل السفر.*	○	○
**مراجعة استخدام الدواء:**<br>*وصف الخدمة: تقييم الحالة الصحية للمريض وتقديم خطة دوائية، ومراقبة الالتزام بالأدوية، والإبلاغ عن الأخطاء الدوائية أو الإبلاغ عن التفاعلات الدوائية الضارة ومنع المشاكل المتعلقة بالأدوية، وتقديم ملاحظات لطبيب المريض حول تقدمه في خطة الرعاية الصيدلانية.*	○	○
**برنامج تثقيف المرضى وتعزيز الصحة:**<br>*وصف الخدمة: نشر الوعي الصحي للمراجعين وتنمية مهارات المجتمع لضمان جودة الحياة صحيا.*	○	○
**خدمات ومرافق اخرى :**	**أعلم عنها<br>أو سمعت عنها**	**لا أعلم عنها<br>أو لم اسمع عنها**
**الوصفات الطبية الإلكترونية (مثلا: نظام وصفتي):**<br>*وصف الخدمة : ربط المراكز الصحية الأولية والمستشفيات(سواء الحكومية او الخاصة) عبر الصيدليات المجتمعية لاستلام أدويتك منها.*	○	○
**توفير السجلات الطبية الالكترونية للمرضى:**<br>*وصف الخدمة: سجل تاريخ المريض لمتابعة حالته المرضية أوالصحية.*	○	○
**توفير الرعاية الصيدلانية من خلال موقع الصيدلية على الانترنت أو الهاتف وتوصيل الأدوية للمنازل:**	○	○
**العيادة الصيدلية :**<br>*وصف الخدمة: لمزيد من الخصوصية يقدم لك الصيدلي الاستشارات والرعاية الصيدلانية المنتضمة في منطقة مخصصة ومغلقة داخل الصيدلية.*	○	○

16. ما مدى أهمية كل خدمة من وجهة نظرك؟

الخدمات الصيدلانية والمرافق ووصف كل خدمة<br><br>**الخدمات الصيدلانية الموسَّعة:**	مهمة جدا	مهمة	محايد	غير مهمة	غير مهمة اطلاقا
**الفحص الصحي:**<br>*وصف الخدمة: برنامج إدارة ومتابعة الوزن ونمط التغذية الصحي. قياس مستوى السكر، أو قياس مستوى ضغط الدم، أو قياس مستوى الكوليسترول، أو قياس نسبة الاكسجين (الحالات الربو). فحص هشاشة العظام وغيرها.*	○	○	○	○	○
**برنامج الإقلاع عن التدخين:**<br>*وصف الخدمة: برنامج متكامل للمدخنين ويشمل ذلك أدوات المساعدة والعلاجات والاستشارات والمتابعة المستمرة.*	○	○	○	○	○
**خدمات التحصين:**<br>*وصف الخدمة: توفير واعطاء اللقاحات، أو لقاحات الانفلونزا الموسمية، أو فحص كوفيد-19.*	○	○	○	○	○
**برنامج صحة المسافر:**<br>*وصف الخدمة: رعاية وقائية، أوعلاجية قبل السفر.*	○	○	○	○	○
**مراجعة استخدام الدواء:**<br>*وصف الخدمة: تقييم الحالة الصحية للمريض وتقديم خطة دوائية، ومراقبة الالتزام بالأدوية، والإبلاغ عن الأخطاء الدوائية أو الإبلاغ عن التفاعلات الدوائية الضارة ومنع المشاكل المتعلقة بالأدوية، وتقديم ملاحظات لطبيب المريض حول تقدمه في خطة الرعاية الصيدلانية.*	○	○	○	○	○
**برنامج تثقيف المرضى وتعزيز الصحة:**<br>*وصف الخدمة: نشر الوعي الصحي للمراجعين وتنمية مهارات المجتمع لضمان جودة الحياة صحيا.*	○	○	○	○	○
**خدمات اخرى ومرافق :**	**مهمة جدا**	**مهمة**	**محايد**	**غير مهمة**	**غير مهمة اطلاقا**
**الوصفات الطبية الإلكترونية (مثلا: نظام وصفتي):**<br>*وصف الخدمة : ربط المراكز الصحية الأولية والمستشفيات(سواء الحكومية او الخاصة) عبر الصيدليات المجتمعية لاستلام أدويتك منها.*	○	○	○	○	○
**توفير السجلات الطبية الالكترونية للمرضى:**<br>*وصف الخدمة: سجل تاريخ المريض لمتابعة حالته المرضية أوالصحية.*	○	○	○	○	○
**توفير الرعاية الصيدلانية من خلال موقع الصيدلية على الانترنت أو الهاتف وتوصيل الأدوية للمنازل.**	○	○	○	○	○
**العيادة الصيدلية :**<br>*وصف الخدمة: لمزيد من الخصوصية يقدم لك الصيدلي الاستشارات والرعاية الصيدلانية المنتضمة في منطقة مخصصة ومغلقة داخل الصيدلية.*	○	○	○	○	○

هـ. موقف المستفيد من توفير الخدمات الموسَّعة في الصيدليات المجتمعية:

والآن، موقفك تجاه هذه الخدمات الموسَّعة ورأيك بشأن الصيادلة المجتمعيون له أهمية بالغة،

17. فضلا أشر الى مدى موافقتك أو معارضتك لكل عبارة من الآتي:

العبارة	أوافق بشدة	أوافق	محايد	أعارض	أعارض بشدة
يمكن للصيدليات المجتمعية توفير خدمة رعاية صيدلانية موسَّعة كباقي المراكز الصحية الأخرى.	◯	◯	◯	◯	◯
الصيادلة المجتمعيون مؤهلون لتقديم الرعاية الصيدلانية الموسَّعة لتحسين مخرجات صحة المجتمع.	◯	◯	◯	◯	◯
أوصي بتنفيذ وتطبيق الخدمات الصيدلانية الموسَّعة في الصيدليات المجتمعية نظرا لأهميتها.	◯	◯	◯	◯	◯
في حال تطبيق بعض برامج الخدمات الصيدلانية الموسَّعة، سأسجل في بعض من هذه البرنامج.	◯	◯	◯	◯	◯
إذا كانت بعض برامج الخدمات الصيدلانية الموسَّعة بمقابل مادي، فسأدفع ثمن هذه الخدمات.	◯	◯	◯	◯	◯
الخدمات الحالية (التقليدية) التي تقدمها الصيدليات المجتمعية تعد كافية لتلبية احتياجات الجمهور.	◯	◯	◯	◯	◯

و. العوائق المحتملة التي قد تمنع الاستفادة من الخدمات الصيدلانية الموسَّعة:

وأخيرا، بعد أن تكونت لديك صورة عن بعض الخدمات الموسَّعة للصيدليات المجتمعية وبينت موقفك تجاهها

18. نودّ أن نعرف رأيك حول العوائق التي قد تمنعك من استخدام هذه الخدمات؟

يمكن اختيار عدة إجابات

☐ عدم القدرة على التواصل الكافي أو المباشر مع الصيدلي. *مثال : نتيجة ازدحام الصيدلية بالمراجعين أو عدم توفر مواعيد سابقة.*

☐ قلة الأماكن المخصصة للاستشارات الشخصية لمناقشة الحالة الصحية داخل الصيدلية أو عدم وجودها (العيادة الصيدلية)

☐ انعدام الثقة بالصيادلة المجتمعيون لتقديم الخدمات الصيدلانية الموسَّعة من خلال الصيدليات المجتمعية.

☐ انعدام التواصل المباشر بين الطبيب والصيدلي لمناقشة حالة المريض الصحية.

☐ لا تسمح الحالة المادية للمريض أو المستهلك بتغطية ثمن بعض الخدمات الصيدلانية .

☐ البنى التحتية للصيدليات المجتمعية والكوادر الصيدلانية المؤهلة غير كافية لتوفير هذه الخدمات حتى الآن.

نهاية الاستبيان.

نقدر لك مشاركتك. فضلا، ساعدنا بنشر هذا الاستبيان من خلال الرابط التالي: https://www.surveymonkey.com/r/3NFCJF8
